# Normative Data for the Swedish Versions of the Beck Anxiety Index and Beck Depression Inventory—Version 2

**DOI:** 10.1002/mpr.70024

**Published:** 2025-08-22

**Authors:** Ann‐Sophie Lindqvist Bagge, Roger Olofsson Bagge, Anders Carlander

**Affiliations:** ^1^ Dept of Psychology, University of Gothenburg Gothenburg Sweden; ^2^ Wallenberg Centre for Molecular and Translational Medicine University of Gothenburg Gothenburg Sweden; ^3^ Department of Surgery Institute of Clinical Sciences Sahlgrenska Academy at the University of Gothenburg Gothenburg Sweden; ^4^ Department of Surgery Sahlgrenska University Hospital Region Västra Götaland Gothenburg Sweden; ^5^ SOM Institute University of Gothenburg Gothenburg Sweden

**Keywords:** anxiety, BAI, BDI‐II, depression, general population, normative, Sweden

## Abstract

**Background:**

Two of the most extensively utilized questionnaires for assessing symptoms of anxiety and depression are Beck Anxiety Inventory (BAI) and Beck Depression Inventory‐second version (BDI‐II).

**Aim:**

Given the absence of normative data based on a representative Scandinavian cohort, the primary objective is to provide normative data from a representative Swedish general population sample and explore potential associations with sociodemographic variables.

**Methods:**

Data was collected 2019 from a pre‐stratified Swedish adult population sample (*n* = 2622).

**Results:**

The average mean value showed: a mild level of anxiety (8.7 ± 9.5) and a minimal level of depression (3.4 ± 5.6). Higher BAI and BDI‐II scores were observed in women and younger individuals. There was an association between educational attainment and BDI‐II scores, where those with a bachelor's degree or higher reported a lower depression score. Results show that BAI and BDI‐II deviate from normality but demonstrate excellent internal consistency (McDonald's omega 0.92 and 0.91, respectively). BAI had a poor model fit (RMSEA) 0.11), while BDI‐II had a fair model fit (RMSEA 0.08).

**Discussion:**

The present study presents normative data for BAI and BDI‐II based on a representative sample from the Swedish general population. The model fit was not ideal, indicating caution, particularly for BAI.

## 1 Introduction

Two of the most widely utilized validated instruments for assessing symptoms of anxiety and depression are the Beck Anxiety Inventory (BAI) (Beck et al. 1988; Piotrowski 2018) and the Beck Depression Inventory‐second version (BDI‐II) (Beck, Steer, Ball, et al. 1996; McDowell 2006)). To make valid and meaningful interpretations of scores derived from self‐report measures like BAI and BDI‐II, it is imperative to contextualize these results with pertinent normative data. The conceptualization, manifestation, and explanation of mental well‐being are shaped by cultural frameworks and local norms (Wang and Gorenstein 2013; American Psychiatric Association 2013). Hence, it holds paramount clinical and scientific significance to provide normative data for mental health assessment instruments based on nationally and culturally representative samples.

Normative data serve as a reference point for determining how a patient or a clinical cohort compares to the expected population. This is particularly crucial when assessing symptom severity between stratified groups or diverse cultures (Mitrushina et al. 2005). Despite the psychometric validity of BDI‐II and BAI (Wang and Gorenstein 2013; Beck and Steer 1993; Leyfer et al. 2006), a limited number of published studies provide normative data for BDI‐II and BAI based on nationally representative samples. To the authors' knowledge, only five English‐written articles for BAI (Crawford et al. 2011; Gillis et al. 1995; Jylha and Isometsa 2006; Magán et al. 2008; Osman et al. 1993) (see Table 1) and six for the BDI‐II (Ciharova et al. 2020; Garcia‐Batista et al. 2018; Ginting et al. 2013; Gomes‐Oliveira et al. 2012; González et al. 2015; Roelofs et al. 2013) (see Table 2) have been published in peer‐reviewed scientific journals presenting normative data based on samples claimed to be representative of national adult populations. Each of these articles varies in research design and sampling methodologies, but generally, some of the studies have a low level of external validity (for further details, see Table 1, Table 2, and Supporting Information S1).

**TABLE 1:** Sample characteristics and psychometric properties of the Beck Anxiety Inventory (BAI) in studies published in English peer‐reviewed scientific journals with samples from general populations.

	Sample characteristics						Psychometrics		
Study	Country	*n*	% of women	Mean age	Sampling method	Administration method	M	SD	*ω* (*α*)
Europe									
Present study	Sweden	2558	48	48,8	Self‐selected	Digital	8.7	9.5	0.92
Jylha and Isometsa 2006	Finland	441	51.2	45.0	Random	Pen and paper	6.3	7.7	(0.91)
Magán et al. 2008	Spain	249	52.6	37.8	Snowball	Digital	11.2	10.3	(0.93)
North America									
Osman et al. 1993	USA	225	70.6	36.9	—	—	11.6	10.3	(0.92)
Gillis et al. 1995	USA	242	—	—	Convenience	—	6.6	8.1	—
Oceania									
Crawford et al. 2011	Australia	729	52.0	41.0	Convenience	Pen and paper	6.2	7.2	(0.90)

**TABLE 2:** Sample characteristics and psychometric properties of the Beck Depression Inventory‐version 2 (BDI‐II) in studies published in English peer‐reviewed scientific journals with samples from general populations.

	Sample characteristics						Psychometrics		
Study	Country	*n*	% of women	Mean age	Sampling method	Administration method	M	SD	*ω* (*α*)
Europe									
Present study	Sweden	2622	48	48.8	Self‐selected	Digital	3.4	5.6	0.91
Roelofs et al. 2013	The Netherlands	7500	57.3	43.3	Random	Digital	10.6	10.9	(0.95)
Ciharova et al. 2020	Czech Republic	450	54.0	—	Self‐selected	—	7.0	5.8	—
North America									
Garcia‐Batista et al. 2018	Dominican Republic	797	—	—	Convenience	Pen and paper	9.1	7.4	—
South America									
Gomes‐Oliveira et al. 2012	Brazil	182	56.0	41.0	Convenience	—	9.9	10.7	(0.93)
González et al. 2015	Mexico	205	51.7	29.9	Convenience		9.8	7.7	(0.87)
Asia									
Ginting et al. 2013	Indonesia	720	—	37.8	Snowball	—	14.2	9.7	—

Due to the absence of scientifically peer‐reviewed articles on normative data derived from representative Scandinavian samples for both BAI and BDI‐II, the primary objective of this study is to provide norm data based on a representative sample obtained from the Swedish general population. Additionally, this study aims to explore potential relationships with sociodemographic factors.

## 2 Methods

### 2.1 Participants

This study had a cross‐sectional research design, and data were obtained via the Swedish citizen panel managed by the SOM Institute at the University of Gothenburg in Sweden (https://www.gu.se/en/som‐institute). The study participants (*N* = 2622) comprised Swedish adult citizens (> 18 years of age) who had previously recruited or enroled in the Swedish citizen panel.

### 2.2 Data Collection Procedure

The Swedish Citizen Panel is a non‐commercial web panel that musters over 75,000 Swedish adult citizens (≥ 18 years of age) recruited by probability‐based samples from the Swedish tax agency register and self‐recruited individuals. Since participants in the Swedish Citizen Panel predominantly consist of males with higher levels of educational attainment compared to the overall Swedish population, invitations were directed toward a pre‐stratified sample of 4000 individuals based on gender, age, and educational attainment to align with the demographic composition of Sweden. Data collection occurred in January 2019. The net response rate reached 65.5%, with a total of 2622 responses gathered over 12 days (BAI: *n* = 2558; BDI‐II: *n* = 2622). A single email reminder was dispatched 7 days after the initial invitation.

### 2.3 Measures

*Beck Anxiety Inventory (BAI)* was initially introduced in 1987 (Beck et al. 1988), with updated cut‐off scores in 1993 (Beck and Steer 1993; Bardhoshi et al. 2016). It comprises 21 items that assess the physiological and cognitive manifestations of anxiety experienced in the past week (including the day of assessment) using a 4‐point scale from 0 (Not at all) to 3 (Severely—I could barely stand it). Scores are summed (total range 0–63), and higher scores indicate an increased intensity of self‐reported anxiety levels (Beck and Steer 1993). According to the score interpretation guidelines for BAI, scores ranging from 0 to 7 signify minimal anxiety, 8–15 indicate mild anxiety, 16–25 represent moderate anxiety, and 26–63 suggest severe anxiety (Beck and Steer 1993; Bardhoshi et al. 2016). BAI is designed for individuals aged 17 years or older and takes 5–10 min to complete (Beck et al. 1988; Leyfer et al. 2006). The English version of BAI has shown satisfactory psychometric properties (Bardhoshi et al. 2016). Pearson Assessment (www.pearsonassessments) holds the copyright for the Swedish translation of BAI (www.pearsonclinical.se/bai). Due to conflicting findings concerning the factor structure for both BAI (Bardhoshi et al. 2016), this research has opted not to present subscales and focuses solely on the total score.

*The Beck Depression Inventory ‐ version 2 (BDI‐II)* (Beck, Steer, Ball, et al. 1996) comprises 21 items that evaluate physiological and cognitive symptoms of depression experienced during the last 2 weeks (including the day of responding) on a 4‐point scale ranging from 0 to 3. BDI‐II scores are summed, and higher scores indicate increased depression levels. Cut‐off scores: 0–13 for minimal depression, 14–19 for mild depression, 20–28 for moderate depression, and 29–63 for severe depression (Beck et al. 1996). The first edition of BDI was developed in 1961 (Beck et al. 1961). In response to the changes in the diagnostic criteria for Major Depressive Disorder presented in the fourth edition of the Diagnostic and Statistical Manual of Mental Disorders (American Psychiatric Association 1994) in 1994, a significant revision was performed in 1996, resulting in the second version of BDI (BDI‐II) (Beck, Steer, Ball, et al. 1996). BDI‐II is designed for individuals aged 13 years or older and takes 10 min to complete. The BDI‐II has demonstrated satisfactory psychometric properties (Wang and Gorenstein 2013; Kjaergaard et al. 2014). The BDI‐II has been observed to discriminate between low levels of depression, unlike some other depression instruments (Wahl et al. 2014). Pearson Assessment (www.pearsonassessments) holds the copyright for the Swedish translation of BDI‐II (www.pearsonclinical.se/bdi‐ii). Due to conflicting findings concerning the factor structure for BDI‐II (Wang and Gorenstein 2013), this research has opted not to present subscales and focuses solely on the total score.

*Demographics and socioeconomic status.* Participants' ages ranged from 18 to 85, and they marked their gender utilizing a three‐point scale (female, male, other). Educational attainment levels were rated on a nine‐point scale ranging from 1 to 2 (elementary school) to 9 (graduate school/Ph.D.). Personal monthly income was assessed on a 13‐point rating scale ranging from 1 (less than 4000 SEK) to 13 (more than 65,000 SEK).

### 2.4 Statistics

We tested each construct (BAI and BDI) for dimensionality and presented the psychometric properties (including McDonald's omega (*ω*) for internal consistency and model fit statistics [root mean square error of approximation (RMSEA), Comparative Fit Index (CFI), Tucker‐Lewis Index (TLI), and Standardized root mean square residual (SRMR)] from a one factor and two factor confirmatory factor analysis (CFA). No additional covariances or error terms beyond the theoretical models were introduced. Normality was tested using a Shapiro‐Francia test (Box‐Cox transformation). We report normative data for total, stratified by men and women across 5 age groups (16–29, 30–39, 40–49, 50–59, 60+) and present range (min/max), mean value (M) including cell‐weighted (sex, age, education) mean in Tables 3, 4, 5, and standard deviation (SD). The software used for all analyses was Stata version 18 (StataCorp 2023).

**TABLE 3:** Overall descriptive statistics for Beck Anxiety Inventory (BAI) and Beck Depression Inventory‐version 2 (BDI‐II).

	Bai	(Cell‐weight)	BDI‐II	(Cell‐weight)
All (*n*)	2558		2622	
Mean ± SD	8.70 ± 9.54	8.79	3.43 ± 5.56	3.37
Median (IQR)	5 (2–12)		1 (0–4)	
Skewness	1.69		2.67	
Kurtosis	6.08		11.49	
Shapiro‐Francia test (*p*)	< 0.001		< 0.001	
Men (*n*)	1311		1342	
Mean ± SD	6.72 ± 8.47	6.83	2.74 ± 4.89	2.73
Median (IQR)	4 (1–9)		1 (0–3)	
Women (*n*)	1204		1234	
Mean ± SD	10.76 ± 10.13	10.89	4.15 ± 6.09	4.06
Median (IQR)	8 (3–15)		2 (0–6)	
≥ Bachelor's degree (*n*)	729		746	
Mean ± SD	8.38 ± 8.63	8.14	2.92 ± 4.65	2.79
Median (IQR)	6 (2–12)		1 (0–4)	
< Bachelor's degree (*n*)	1789		1833	
Mean ± SD	8.80 ± 9.85	9.09	3.62 ± 5.86	3.60
Median (IQR)	5 (2–13)		1 (0–4)	

**TABLE 4:** Normative data (number of participants, range, mean, and standard deviations) on the Beck Anxiety Inventory (BAI) stratified by sex and age groups.

Sex	Age group	*n*	Min	Max	M	M (cell‐weight)	SD
Males	16–29	138	0	47	10.38	11.22	10.55
	30–39	230	0	55	8.22	7.74	8.82
	40–49	261	0	46	6.48	6.87	8.09
	50–59	233	0	51	6.70	7.42	9.36
	60+	390	0	40	4.90	4.69	6.54
Females	16–29	144	0	46	14.70	14.07	10.39
	30–39	223	0	58	14.58	14.78	11.80
	40–49	199	0	48	10.55	10.81	9.86
	50–59	246	0	54	10.43	10.24	10.13
	60+	305	0	44	6.78	7.06	6.88

**TABLE 5:** Normative data (number of participants, range, mean, and standard deviations) on Beck Depression Inventory‐version 2 (BDI‐II) stratified by sex and age groups.

Sex	Age group	*n*	Min	Max	M	M (cell‐weight)	SD
Males	16–29	145	0	31	4.01	3.90	5.85
	30–39	237	0	37	3.45	3.01	5.58
	40–49	267	0	31	2.94	3.29	5.31
	50–59	235	0	32	2.79	3.15	5.28
	60+	398	0	27	1.82	1.70	3.36
Females	16–29	150	0	32	5.11	4.80	5.89
	30–39	227	0	41	5.91	5.54	7.58
	40–49	205	0	31	3.62	3.71	5.14
	50–59	249	0	37	4.69	4.51	7.10
	60+	311	0	29	2.43	2.65	4.15

## 3 Ethical Approval

The study was approved by the Swedish Ethical Review Authority (Dnr: 948–18). Written informed consent was obtained from all participants.

## 4 Results

### 4.1 Participant Characteristics

Approximately an equal number of males (51.8%) and females (48.2%) were present. The participants reported a mean age of 48.8 ± 15.4 years, and almost a third (28.7%) reported holding a bachelor's degree or higher. These figures can be compared approximately to the official numbers from Statistics Sweden for 2019 (www.scb.se/en/): males 50.3%, mean age 41.3, bachelor's degree or higher ≈ 23% (note: age 16–94+).

### 4.2 Normative Data ‐ BAI

The average score of BAI suggests mild anxiety (8.70–9.54) compared to the clinical cut‐off scores (Beck and Steer 1993; Bardhoshi et al. 2016) (Table 3). The observed total mean score of BAI is consistent with previous findings from comparable studies (Table 1). The mean BAI score was significantly *t* (2513) = 10.87, *p* < 0.0001 higher among women compared to men (*d* ≈ 0.43), but did not significantly (*p* = 0.310) differ regarding educational attainment. Age was significantly and inversely correlated with anxiety, showing that higher age was associated with lower BAI scores (*r* = −0.24, *p* < 0.001). See Table 3. Normative data stratified by sex and age groups on BAI are presented in Table 4.

In terms of normality, the level of skewness and kurtosis in BAI differed significantly (*p* < 0.001) from normality when conducting a Shapiro‐Francia test using the Box‐Cox transformation. Applying a post‐stratification cell weight on age, sex, and education on the test for normality described by Royston (Royston 1991) does not affect the indication that BAI is normally distributed in the present sample (*p* < 0.001). In terms of model fit, a one factor confirmatory factor analysis (CFA) indicates that the model fit for the Beck's Anxiety Inventory (BAI) is poor (Root Mean Square Error of Approximation (RMSEA): 0.11, Comparative Fit Index (CFI): 0.76, Tucker‐Lewis Index (TLI): 0.73), albeit that the Standardized Root Mean Square Residual (SRMR): 0.07 demonstrate a good model fit. The common two factor model (somatic vs. cognitive) reported by Beck et al. 1988 indicates a relatively better fit (Root Mean Square Error of Approximation (RMSEA): 0.09, Comparative Fit Index (CFI): 0.84, Tucker‐Lewis Index (TLI): 0.82), Standardized Root Mean Square Residual (SRMR): 0.06. See Table 1 for an analysis of the internal consistency comparing our estimate (*ω*) with earlier studies (*α*). The observed alpha coefficient aligns with prior published results based on similar samples.

### 4.3 Normative Data ‐ BDI‐II

As can be seen in Table 3, the total mean score of BDI‐II corresponds to clinically minimal depression (3.43 ± 5.56) (Beck et al. 1996). The observed total mean score of BDI‐II is lower than prior published results based on similar samples (Table 2).

BDI‐II was significantly *t* (2574) = 10.87, *p* < 0.0001 higher among women than men (*d* ≈ 0.26). There was also a significant *t* (2577) = 2.90, *p* = 0.004 difference between educational attainment groups where participants with a bachelor's degree or higher reported a lower depression score (*d* ≈ 0.13). The results also indicate that depression is inversely correlated to age (*r* = −0.16, *p* < 0.001). See Table 3. Normative data stratified by sex and age groups on BDI‐II are presented in Table 5.

Level of skewness and kurtosis in BDI‐II differ significantly (*p* < 0.001) from normality when conducting a Shapiro‐Francia test using the Box‐Cox transformation. Applying a post‐stratification cell weight on age, sex, and education on the test for normality described by Royston (Royston 1991) does not affect the indication that BDI‐II is normally distributed in the present sample (*p* < 0.001). In contrast, a CFA for the BDI‐II shows that model fit for a one factor model is near adequate for most indices (Root Mean Square Error of Approximation (RMSEA): 0.08, Comparative Fit Index (CFI): 0.84, Tucker‐Lewis Index (TLI): 0.83, Standardized Root Mean Square Residual (SRMR): 0.05), but improved in the two factor model of somatic vs. cognitive (e.g., Beck et al. 1996) (Root Mean Square Error of Approximation (RMSEA): 0.06, Comparative Fit Index (CFI): 0.91, Tucker‐Lewis Index (TLI): 0.89, Standardized Root Mean Square Residual (SRMR): 0.04). See Table 2 for an analysis of the internal consistency comparing our estimate (*ω*) with earlier studies (*α*). Cutoff values for internal consistency are arbitrary, but clinical applications should be more conservative, and values above 0.90 may indicate a high degree of reliability within the instrument.

## 5 Discussion

As of today, we provide the largest population‐based norm data for two of the most used instruments for measuring anxiety and depression, namely the BAI and the BDI‐II. This study presents normative data for BAI and BDI‐II based on a representative sample of the Swedish general population. Given the great cultural similarities between the Scandinavian countries, the current result may possibly act as normative data for all Scandinavian countries.

Concerning BAI, the present results showed a reported mild anxiety among the Swedish adult population (total score of 8.7 ± 9.5). Five comparably English‐written studies presenting normative data based on samples claimed to be representative of national adult populations have been published in peer‐reviewed scientific journals (Crawford et al. 2011; Gillis et al. 1995; Jylha and Isometsa 2006; Magán et al. 2008; Osman et al. 1993) (Table 1). The measured average is slightly higher than the normative BAI results from Finland (+2.4) (Jylha and Isometsa 2006) and Australia (+2.5) (Crawford et al. 2011), but lower than Spain (−2.5) (Magán et al. 2008), while studies from the USA showed a higher average (+2.1) (Gillis et al. 1995) but also lower (−2.9) (Osman et al. 1993). It is difficult to explain the observed differences in total scores for the BAI, but large differences exist in recruitment methods between the different studies. From a cultural perspective, it should be noted that although the scattered results reflect national differences in the population's perceived anxiety level during data collection, all studies are from Western cultures.

The present demographic data shows that Swedish women score significantly higher on symptoms of anxiety compared to Swedish men, 10.8 ± 10.1 (mild anxiety) compared to 6.7 ± 8.5 (minimal anxiety). This result is in line with other BAI‐normative studies from Finland (Jylha and Isometsa 2006), the USA (Osman et al. 1993), and Spain (Magán et al. 2008) but contradicts other studies from the USA (Gillis et al. 1995) and Australia (Crawford et al. 2011) reporting no significant gender difference or weak gender correlation. The present results showed that the younger Swedish population scored higher on anxiety symptoms than the older Swedish population, which is in parallel with normative data from the USA (Gillis et al. 1995; Osman et al. 1993). However, studies from Spain (Magán et al. 2008) and Australia (Crawford et al. 2011) showed no significant or weak correlation with age. Educational attainment did not affect the anxiety level of the Swedish population, which is in line with normative data from Spain (Magán et al. 2008) and Australia (Crawford et al. 2011).

Beyond the mostly expected negative skew from this normal population, the internal consistency indicated a high degree of reliability on the BAI instrument. In contrast, the CFA demonstrated a relatively poor model fit. These deviations may be attributed to the skew and non‐normality in the data or to the fact that the BAI instrument may be less reliable in a normal population cohort. It should also be acknowledged that most previous studies reporting normative data on the BAI instrument do not report model fit statistics from a CFA.

Concerning Swedish normative data for BDI‐II, the present results showed that the total score was 3.4 ± 5.6, indicating a self‐rated minimal depression. Six comparably English‐written studies have been identified for presenting normative data on BDI‐II based on samples claimed to be representative of national adult populations published in peer‐reviewed scientific journals (Ciharova et al. 2020; Garcia‐Batista et al. 2018; Ginting et al. 2013; Gomes‐Oliveira et al. 2012; González et al. 2015; Roelofs et al. 2013) (Table 2). The measured average is lower than the published normative BDI‐II results from countries in Europe (The Netherlands −7.2 (Roelofs et al. 2013); Czech Republic −3.6 (Ciharova et al. 2020), North America (−5.7) (Garcia‐Batista et al. 2018) and South America (Brazil −6.5 (Gomes‐Oliveira et al. 2012); Mexico −6.4 (González et al. 2015)), and Asia (Indonesia −10.8) (Ginting et al. 2013). It is challenging to provide explanations for the observed lower total scores for symptoms of depression in Sweden. A possible explanation for the observed lower BDI‐II scores could be that the participants had a slightly higher mean age, level of education, and proportion of men compared to the Swedish population. It has previously been shown that male gender (Albert 2015) and older age (Sikstrom et al. 2023) are correlated with lower self‐rated depression symptoms.

The present demographic data shows that Swedish adult women score significantly higher on symptoms of depression compared to Swedish adult men. This result is consistent with previous results reporting higher BDI‐II depression scores reported by women than men (Ciharova et al. 2020; González et al. 2015; Roelofs et al. 2013). The present results showed that the younger Swedish population scored higher on depression symptoms than the older Swedish population, contradicting normative BDI‐II studies from the Netherlands (Roelofs et al. 2013) and Mexico (González et al. 2015), showing no impact of age on BDI‐II scores. Educational attainment did have an impact on reported symptoms of depression in the Swedish population, where participants with a bachelor's degree or higher reported a lower depression score. This result aligns with data from the Netherlands (Roelofs et al. 2013) but contradicts data from the Czech Republic (Ciharova et al. 2020), reporting no difference in BDI‐II scores depending on educational attainment.

Like the BAI instrument, the internal consistency of the BDI‐II instrument was excellent. However, in contrast to the BAI instrument, the BDI‐II instrument rendered an overall better—albeit not good—model fit from the CFA. This could indicate that the BDI‐II instrument is more suitable for non‐clinical populations than the BAI instrument. Yet, the BDI‐II instrument demonstrates a more severe deviation from normality where there is more positive skew, meaning that most scores are low, but a few are higher than expected. In addition, the level of kurtosis is also higher in the BDI‐II instrument, which indicates more extreme tail values.

### 5.1 Limitations

This study is not without limitations. The following should specifically be noted. Normative data is ideally derived from a random probability sample. This study, however, employs a more resource‐efficient approach by using a pre‐stratified self‐selected sample that aligns with the Swedish population. Despite attempts to completely match the Swedish demographics, the participants were, on average, slightly older and more educated, and the data included a higher proportion of men, which may have influenced the results. The data underwent pre‐stratification for gender, age, and educational attainment, ensuring alignment with Sweden's demographic composition. Of course, factors beyond gender, age, and educational attainment may affect self‐reported levels of anxiety and depression, thereby introducing a potential unknown bias in the sample selection.

## 6 Conclusions

As of today, we provide the largest population‐based norm data for two of the most used instruments for measuring anxiety and depression, namely the BAI and the BDI‐II. The result showed scores corresponding to self‐rated mild anxiety and minimal depression among the Swedish adult population.

Demographic analysis observed higher levels of anxiety and depressive scores in women and younger individuals, and educational attainment did have an impact on reported symptoms of anxiety in the Swedish population. There was, however, an educational attainment effect for BDI‐II, where Swedish citizens with a bachelor's degree or higher reported a lower depression score. The BAI and BDI‐II deviate from normality but demonstrate excellent internal consistency (McDonald's omega 0.92 and 0.91, respectively). BAI had a poor model fit (RMSEA 0.11), while BDI‐II had a fair model fit (RMSEA 0.08), which should warrant caution when using these tools in normal populations.

## Author Contributions

**Ann‐Sophie Lindqvist Bagge:** conceptualization, investigation, methodology, project administration, resources, supervision, validation, visualization, writing – original draft preparation, writing – review and editing. **Roger Olofsson Bagge:** conceptualization, funding acquision, investigation, methodology, resources, validation, visualization, writing – original draft preparation, writing – review and editing. **Anders Carlander:** conceptualization, data curation, formal analysis, investigation, methodology, resources, supervision, validation, visualization, writing – original draft preparation, writing – review and editing.

## Ethics Statement

The study was approved by the Swedish Ethical Review Authority (Dnr: 948–18).

## Consent

Electronic informed consent was obtained from all participants.

## Conflicts of Interest

The author ROB has received institutional research grants from Bristol‐Myers Squibb (BMS), Endomagnetics Ltd (Endomag), SkyLineDx and NeraCare GmbH, speaker honorarium from Roche, Pfizer and Pierre‐Fabre, and has served on advisory boards for Amgen, BD/BARD, Bristol‐Myers Squibb (BMS), Cansr. com, Merck Sharp and Dohme (MSD), Novartis, Roche and Sanofi Genzyme, and is a shareholder in SATMEG Ventures AB. None of the work is linked in any way to the current manuscript. The authors declare that the research was conducted in the absence of any commercials or financial relationships that in any way could be construed as a potential conflict of interest.

## Permission to Reproduce Material From Other Sources

The authors have nothing to repor.

## Supporting information

Supporting Information S1

## Data Availability

Due to the SOM Institute's policy, data cannot be shared publicly. However, the authors can make the data available to researchers who meet the criteria for access to confidential data.
