# Supplementary material for: Normative Data for the Swedish Versions of the Beck Anxiety Index and Beck Depression Inventory—Version 2
Source: Int J Methods Psychiatr Res. 2025 Aug 22;34(3):e70024. doi: 10.1002/mpr.70024 (PMC12371443; doi:10.1002/mpr.70024)
Supplement: Supplementary file 1 — Supporting Information S1 [file MPR-34-e70024-s001.docx]

***S*upplementary text**

Regarding BAI, the authors have identified five articles written in English and published in peer-reviewed scientific journals that present normative data based on samples claimed to be representative of national adult populations. The first published article by Osman et al 1993 ^1^ was based on a sample (n=225) of a general US (20-74 yrs) population. Participants were recruited from a moderate-sized Midwestern community (data on representativeness, mode of administration, exclusion, and inclusion criteria, as well as drop-out/withdrawal rates, were not otherwise stated). The second published article by Gillis et al 1995 ^2^ was based on a sample (n=242) of a general US (18-65 yrs.) population representative regarding sex, race, income, and age. Participants were approached by representatives of two marketing firms at two shopping malls, a Catholic church, a trailer park, and a homeless shelter, all located in the suburbs outside Washington, DC. The exclusion criteria were participants under 18 or older than 65 years or who fit a sociodemographic category (sex, race, income, age) for which the quota had already been reached. Participants individually answered the BAI. The mode of administration and return of the questionnaire, nor drop-out/withdrawal rates, were specifically stated. The third published article by Jylhä & Isometsä, 2006 ^3^ was based on a random sample (n=441) of a general Finnish adult population. The recruitment and data collection took place in two adjacent cities (Espoo and Vantaa, with a combined population of 408,270) in 2003. Nine hundred participants were randomly drawn from the Population Register Center in Finland. Participants digitally completed the BAI. The fourth article by Magán & García-Vera 2008 ^4^ was based on a sample (n=249) of a general Spanish population (18-78 yrs.) representative in age and gender. The participants were recruited through the “snowball” technique, during which university psychology students were asked to invite their relatives and friends to participate in a study. The participants were individually administered the BAI (the mode of administration and return of the questionnaire was not specifically stated). Thirteen participants were discarded due to incompletely answered questionnaires or those who did not indicate their sex or age. Other descriptions of drop-out/withdrawal rates were not stated. The fifth article by Crawford et al 2011 ^5^ was based on a sample (n=785) of the general Australian adult population representative regarding age, education, and gender. The recruitment and data collection occurred in Adelaide, South Australia, between 1995 and 2000. The participants were recruited through various sources, such as local and national businesses, public service organizations, community centers, and recreational groups. Participants completed the BAI by pen and paper, and the questionnaires were either collected later by the investigators or returned by the participants by mail. The combined rate of refusals, non-returns, and incomplete returns was 28.5%.

Concerning BDI-II, the authors believe that six articles written in English presenting normative data based on samples claimed to be representative of national adult populations have been published in peer-reviewed scientific journals. The first study by Gomes-Oliveira et al 2012 ^6^ was based on a community-dwelling sample of the general Brazilian adult population. A convenience sample of n=182 adult participants from the city of São Paulo was drawn from a population-based household survey (year, further information on data collection, and mode of administration were not stated). The second study by Roelof et al 2013 ^7^ was based on a sample of the general Dutch adult population (18–65 yrs.). The Dutch municipalities provided a random selection of 217,816 names and addresses of Dutch residents who received an invitation letter to complete a screening questionnaire via the Internet. 7,500 participants digitally completed the BDI-II (year not stated). The third study by Ginting et al 2013 ^8^ was based on n=720 Indonesians recruited randomly using snowball sampling techniques. Apart from being reported to be mentally and physically healthy, no further information was given on data collection and mode of administration. The fourth study by Gonzáles et al 2015 ^9^ was based on a community sample (n=205) of Mexican residents living in Mexico City. The residents were selected by convenience and approached in different public parks and plazas in Mexico City. They were asked to participate in the study (further information on data collection and mode of administration was not stated). The fifth study by Garcia-Batista et al 2018 ^10^ was based on a sample (n=797) from the Dominican Republic selected by convenience from the general population (no further information about the selection of participants was stated). A paper version of the BDI-II was administered by a suitably trained team (no further information about the mode of administration was stated). The sixth study by Ciharova et al 2020 ^11^ was collected in 2016 using a cross-sectional design. Participants were community residents (n=450, 18-96 yrs.) recruited through convenience, non-random quota (defined as regions) sampling in all 14 regions of the Czech Republic. The sample was regionally representative and was obtained via advertisements on the website of the General University Hospital in Prague and the Prague School of Psychosocial Studies. Exclusion criteria were: age < 18 years, uncorrected visual or hearing impairment, major depression or other major psychiatric disorder, a history of alcohol or substance abuse, epilepsy, previous head injury resulting in unconsciousness, any neurodegenerative disease, stroke, undergoing radiotherapy or chemotherapy and unstable medical illness. Out of 616 invited participants, N=450 participants were included in the final sample and completed the BDI-II (mode of administration was not specifically stated).

1 Osman, A., Barrios, F. X., Aukes, D., Osman, J. & Markway, K. The Beck Anxiety Inventory: Psychometric Properties in a Community Population. *Journal of Psychopathology and Behavioral Assessment* **15**, 287-297 (1993). <https://doi.org:10.1007/BF00965034>

2 Gillis, M. M., Haaga, D. A. F. & Ford, G. T. Normative Values for the Beck Anxiety Inventory, Fear Questionnaire, Penn State Worry Questionnaire, and Social Phobia and Anxiety Inventory. *Psychological Assessment* **7**, 450-455 (1995). <https://doi.org:10.1037/1040-3590.7.4.450>

3 Jylha, P. & Isometsa, E. The relationship of neuroticism and extraversion to symptoms of anxiety and depression in the general population. *Depression and anxiety* **23**, 281-289 (2006). <https://doi.org:10.1002/da.20167>

4 Magán, I., Sanz, J. & García-Vera, M. P. Psychometric Properties of a Spanish Version of the Beck Anxiety Inventory (BAI) in General Population. *The Spanish Journal of Psychology* **11**, 626-640 (2008). <https://doi.org:10.1017/S1138741600004637>

5 Crawford, J., Cayley, C., Lovibond, P. F., Peter H Wilson, P. H. & Hartley, C. Percentile Norms and Accompanying Interval Estimates from an Australian General Adult Population Sample for Self‐Report Mood Scales (BAI, BDI, CRSD, CES‐D, DASS, DASS‐21, STAI‐X, STAI‐Y, SRDS, and SRAS). *Australian Psychologist* **46**, 3-14 (2011). <https://doi.org:10.1111/j.1742-9544.2010.00003.x>

6 Gomes-Oliveira, M. H., Gorenstein, C., Lotufo Neto, F., Andrade, L. H. & Wang, Y. P. Validation of the Brazilian Portuguese version of the Beck Depression Inventory-II in a community sample. *Braz J Psychiatry* **34**, 389-394 (2012). <https://doi.org:10.1016/j.rbp.2012.03.005>

7 Roelofs, J. *et al.* Norms for the Beck Depression Inventory (BDI-II) in a Large Dutch Community Sample. *Journal of psychopathology and behavioral assessment* **35**, 93-98 (2013). <https://doi.org:10.1007/s10862-012-9309-2>

8 Ginting, H., Näring, G., van der Veld, W. M., Srisayektic, W. & Becker, E. S. Validating the Beck Depression Inventory-II in Indonesia’s general population and coronary heart disease patients. *International Journal of Clinical and Health Psychology* **13**, 235−242 (2013). <https://doi.org:10.1016/S1697-2600(13)70028-0>

9 González, D. A., Rodríguez, A. R. & Reyes-Lagunes, I. Adaptation of the BDI–II in Mexico. *Salud Mental* **38**, 237-244 (2015). <https://doi.org:10.17711/SM.0185-3325.2015.033>

10 Garcia-Batista, Z. E., Guerra-Pena, K., Cano-Vindel, A., Herrera-Martinez, S. X. & Medrano, L. A. Validity and reliability of the Beck Depression Inventory (BDI-II) in general and hospital population of Dominican Republic. *PloS one* **13**, e0199750 (2018). <https://doi.org:10.1371/journal.pone.0199750>

11 Ciharova, M., Cigler, H., Dostalova, V., Sivicova, G. & Bezdicek, O. Beck depression inventory, second edition, Czech version: demographic correlates, factor structure and comparison with foreign data. *Int J Psychiatry Clin Pract* **24**, 371-379 (2020). <https://doi.org:10.1080/13651501.2020.1775854>
